# Ledoyen’s Disinfecting Fluid

**Published:** 1847-12

**Authors:** 


*Ledoyen's Disinfecting Fluid.*—This article appears to be exciting considerable attention abroad. We gather from various journals, and particularly the *Pharmaceutical Journal*, for August, 1847, that M. Ledoyen, the discoverer, claims for it all the efficacy usually attributed to chloride of lime, as a disinfectant, without the disadvantage of causing the evolution of any deleterious compound, and that night soil or other refuse, might, by means of this fluid, be entirely deprived of its noxious odour, at the same time, that its properties as a manure would be rather improved than deteriorated. It was also described as being a certain specific against contagion, and an unexpensive compound, easily procurable.

M. Ledoyen refused to disclose his secret, on the ground that in doing so, he would deprive himself of the chance of any pecuniary advantage, although he at the same time offered to submit the liquid to experiment before any tribunal.

M. Ledoyen, however, in conjunction with Col. Calvert, succeeded in introducing the fluid to the notice of the government; a parliamentary inquiry ensued, and a report was published. During the whole of these proceedings, the secret was kept so inviolate, that none of the fluid could be obtained, except on a promise that it should not be chemically examined, and we could not even obtain it from the Fever Hospital, where it had been in use for many weeks, because we declined to take the pledge. A small quantity, however, was recently furnished from another source without the pledge.

It is now generally known that the fluid is a solution of nitrate of lead, which, like other salts of lead, decomposes sulphuretted hydrogen and hydrosulphate of ammonia, thus destroying the smell and producing innoxious compounds. That the salts of lead possess this property is known to every student in chemistry, and these salts are familiar to the profession as applications in the form of lotion and for other purposes. We have known Mr. Ure prescribe a solution of nitrate of lead as a lotion for foul ulcers two or three years ago.

It therefore requires no further proof to establish the fact, that the salts of lead are adapted as decomposing agents, for neutralizing animal or vegetable effluvia, in which sulphuretted hydrogen is the active ingredient. But it remains to be proved that the fluid in question is a specific against the miasma of fever, which, in the opinion of some of our best chemical and medical authorities, is distinct from sulphuretted hydrogen, although generally a concomitant.

On the supposition that sulphuretted hydrogen is the cause of fever, it might be expected that the students in a chemical laboratory, where the air is constantly contaminated with the gas, would be particularly liable to febrile attacks. This, however, is not found to be the case.

It appears that M. Ledoyen does not contemplate securing to himself by patent the exclusive right to sell his fluid, but claims from the government, such reward as may appear on investigation to be due to him, as the discoverer of a disinfecting agent of great importance to the health and welfare of mankind. A similar application was made to the French government, but the grant was refused.—*Pharm. Journ.*, August 1847. T. R. B.

